# LPS Structure and PhoQ Activity Are Important for *Salmonella* Typhimurium Virulence in the *Gallleria* *mellonella* Infection Model

**DOI:** 10.1371/journal.pone.0073287

**Published:** 2013-08-08

**Authors:** Jennifer K. Bender, Thorsten Wille, Kathrin Blank, Anna Lange, Roman G. Gerlach, Junior Research Group

**Affiliations:** Robert Koch-Institute, Wernigerode Branch, Wernigerode, Germany; KIIT University, India

## Abstract

The larvae of the wax moth, *Galleria* *mellonella* , have been used experimentally to host a range of bacterial and fungal pathogens. In this study we evaluated the suitability of *G* *. mellonella* as an alternative animal model of *Salmonella* infection. Using a range of inoculum doses we established that the LD_50_ of *Salmonella* Typhimurium strain NCTC 12023 was 3.6 × 10^3^ bacteria per larva. Further, a set of isogenic mutant strains depleted of known virulence factors was tested to identify determinants essential for *S* . Typhimurium pathogenesis. Mutants depleted of one or both of the type III secretion systems encoded by *Salmonella* Pathogenicity Islands 1 and 2 showed no virulence defect. In contrast, we observed reduced pathogenic potential of a *phoQ* mutant indicating an important role for the PhoPQ two-component signal transduction system. Lipopolysaccharide (LPS) structure was also shown to influence *Salmonella* virulence in *G* *. mellonella* . A *waaL* (*rfaL*) mutant, which lacks the entire O-antigen (OAg), was virtually avirulent, while a *wzz*_ST_/*wzz*_fepE_ double mutant expressing only a very short OAg was highly attenuated for virulence. Furthermore, shortly after infection both LPS mutant strains showed decreased replication when compared to the wild type in a flow cytometry-based competitive index assay. In this study we successfully established a *G* *. mellonella* model of *S* . Typhimurium infection. By identifying PhoQ and LPS OAg length as key determinants of virulence in the wax moth larvae we proved that there is an overlap between this and other animal model systems, thus confirming that the *G* *. mellonella* infection model is suitable for assessing aspects of *Salmonella* virulence function.

## Introduction

*Salmonella enterica* is one of the most prevalent bacterial pathogens worldwide. The impressive success of this pathogen can be attributed to its great versatility in surviving within the environment and the capacity to infect a wide range of host organisms [1]. Key to these processes are the array of factors encoded within the genome, which facilitate host colonization and the development of disease [2]. In order to identify novel virulence determinants that represent putative targets for drug development, researchers have designed numerous models of infection based on cell cultures or entire host organisms. Murine models have been used extensively to study the interactions occurring between host and a variety of bacterial pathogens, and the infection of mice with *Salmonella enterica* serovar Typhimurium ( *S* . Typhimurium ) has been used for decades as a disease model of human typhoid fever. Subsequent refinements to this system allowed for the induction of *Salmonella* -mediated colitis by pretreatment of these animals with streptomycin [3], while genetic modifications of the host have resulted in models that are a much closer representation of *Salmonella* Typhi infection in humans [4,5]. Although these model systems help to improve our understanding of *Salmonella* pathogenesis, infection studies using mammals are often time consuming and require expensive experimental setup. Furthermore, animal numbers must be kept to a minimum and extensive demands have to be fulfilled to meet the high standards given by law and ethics. Therefore, alternative *in vivo* models that avoid these limitations would benefit our ability to analyze the complex interplay between a pathogen and a multi-cellular host organism.

In recent years, substantial progress has been made in developing invertebrate-based model systems for studying bacterial and fungal infections [6–8]. It is worth stressing that a suitable surrogate for mammals ought to give comparable results if the gene or pathway of interest is of any importance for human infection. In the case of the soil nematode *Caenorhabditis elegans*, it has been demonstrated that an alternative sigma factor of *Pseudomonas aeruginosa* is required for virulence in this invertebrate and mice alike [9]. In addition, *Salmonella* virulence determinants that similarly affect *C. elegans* intestinal cells and mammalian epithelial cells have also been described [10]. A key virulence factor of *Salmonella* is the lipopolysaccharide (LPS) or endotoxin, a complex protruding structure connected to the outer membrane of Gram-negative bacteria via a phospholipid anchor, the Lipid A. Attached to this are repetitive sequences of sugar moieties constituting the core oligosaccharide and the O-antigen (OAg), a polysaccharide structure of variable length. Although LPS is highly immunogenic and activates innate immune defense pathways within the host it also mediates complement resistance and interferes with phagocytosis, and is therefore a prerequisite for full virulence in mice [11,12]. Using a nematode model of infection, Aballay et al. showed that an intact bacterial cell envelope containing full-length LPS is required to establish persistent *Salmonella* infection of the *C. elegans* intestine [13]. Similarly, expression of OAg by the apathogenic *E. coli* strain, K-12, rendered this organism fully virulent in the same model, underlining the importance of this surface structure to the colonization of an invertebrate host [14].

Another well-established alternative model organism is the greater wax moth, *Galleria* *mellonella* . *G* *. mellonella* larvae offer considerable benefits as an infection model as they are easy to handle, rapidly generate data and can be studied in large numbers while keeping experiments cost-effective. Most importantly, and in contrast to many other invertebrate models, including *C. elegans*, analyses can be performed at 37° C, a temperature optimal for the vast majority of human pathogens. Moreover, injection of bacteria into the larval hemolymph enables the application of a defined bacterial dose, an advantage over the imprecise infection of *C. elegans*, which relies on the worms freely grazing on a lawn of bacteria to establish colonization. A further advantage of this model is the innate immune response of *G* *. mellonella* , which shares a high degree of homology with the mammalian system [15]. For example, hemocytes, which produce a robust oxidative burst, behave similarly to phagocytic cells in response to bacterial infection [16]. This, amongst other conserved features of anti-microbial action, might explain the positive correlation between data obtained from *Galleria* and mice infections for both eukaryotic pathogens, such as *Candida albicans* [17] or *Aspergillus fumigatus* [18], as well as for different prokaryotes. Hence, the *G* *. mellonella* model has been successfully applied to assess the virulence of a variety of bacteria, including: *Listeria monocytogenes* [19], *Francisella tularensis* [20], *Burkholderia cepacia* complex [21], *P. aeruginosa* [22,23], *Cryptococcus neoformans* [24], *Enterococcus faecium* [25], *Legionella pneumophila* [26] and various corynebacteria [27]. In contrast, data regarding the pathogenicity of *S* . Typhimurium for *G* *. mellonella* is scarce. Although Krustak and colleagues initially described the cellular response of the larvae upon *Salmonella* challenge and bacteria-mediated lysis of the hemocytes, a detailed examination of the responsible determinants was not pursued [28]. Thus, the mechanism behind *Galleria-Salmonella* interaction remains obscure. Here, we set out to establish a *G* *. mellonella* -based model system for studying *Salmonella* virulence, which could be used to generate indicatory data prior to comprehensive mammalian studies.

## Materials and Methods

### Cloning

All bacterial strains and plasmids used in this study are listed in Table 1. An overview about the oligonucleotides used is given in Table 2. The gene for Superfolder GFP (SFGFP) [29] was synthesized codon-optimized for expression in *S. enterica* (Geneart, Regensburg, Germany). The *sfgfp* gene was amplified by PCR using primers SmaI-RBS-SFGFP-for and SFGFP-NcoI-rev and the product was cloned via *Sma*I/*Nco*I in the similarly-digested pWRG15 [30], yielding pWRG81. The EM7 promoter (Life Technologies) was amplified by PCR from pGEN-luxCDABE [31] using primers EcoRI-EM7-for and EM7-rev and then cloned into pWRG81 via *Eco*RI/*Sma*I, yielding pWRG167. The photo-stable TagRFP variant, TagRFP-T [32], was synthesized codon-optimized for expression in *S. enterica* (Geneart). The gene encoding TagRFP-T was amplified by PCR using primers XbaI-TagRFP-for and TagRFP-HindIII-rev and the product was cloned via *Xba*I/*Hind*III into the similarly-digested pFPV25.1 [33], yielding pWRG435.

**Table 1:** Bacterial strains and plasmids used in this study.

*Salmonella* strain	Relevant characteristic(s)	Source or Reference
NCTC 12023	wild type, Nal^S^, isogenic to ATCC 14028	NCTC, Colindale, UK
MvP724	Δ*wzz*_ST_ FRT Δ*wzz*_fepE_ FRT	[42]
MvP818	Δ*invC* FRT	[60]
MvP1036	Δ*waaL* (*rfaL*) FRT	[61]
MvP1213	Δ*fliI* FRT	M. Hensel, unpublished
P2D6	*ssaV*::mTn*5*, Km^r^	[50]
WRG6	Δ*phoQ*	[62]
WRG107	Δ*invC* FRT *ssaV*::mTn*5*, Km^r^	This study
Plasmids		
p3313	P_*rfaD*_::*rfaDFCL* in pWSK29, Ap^r^	[61]
p3390	*wzz*_ST_ and *wzz*_fepE_ in pWSK29, Ap^r^	[42]
pFPV25.1	P_*rpsM*_::*gfpmut3a* in pFPV25, Ap^r^	[33]
pWSK29	Low copy-number vector, Ap^r^	[63]
pWRG81	*sfgfp* in pWRG15 [30], Ap^r^	This study
pWRG103	P_*phoP*_::*phoPQ* in pWSK29, Ap^r^	[62]
pWRG167	P_*EM7*_::*sfgfp* in pWRG81, Ap^r^	This study
pWRG435	P_*rpsM*_::*tagrfp-t* in pFPV25, Ap^r^	This study

**Table 2:** Oligonucleotides used in this study.

Oligonucleotide	Sequence (5’→3’), restriction sites underlined
EcoRI-EM7-for	CAGG A A T T CATCCGCGGCCGCGTTTAAAC
EM7-rev	AGAGGATCC C C G G GTACCAC
SFGFP-NcoI-rev	ATAC C A T G GTTATTATTTATACAGTTCATCCATG
SmaI-RBS-SFGFP-for	ATCC C C G G GAAAGAGGAGAAAAGTATGCGCAAAGGCGAAGAACTG
TagRFP-HindIII-rev	GCGA A G C T TATTATTTATACAGTTCATCCATGC
XbaI-TagRFP-for	GCGT C T A G ATTTAAGAAGGAGATATACATATGGTGAGCAAAGGCGAAGAACTG
SmaI-RBS-SFGFP-for	ATCC C C G G GAAAGAGGAGAAAAGTATGCGCAAAGGCGAAGAACTG
TagRFP-HindIII-rev	GCGA A G C T TATTATTTATACAGTTCATCCATGC
XbaI-TagRFP-for	GCGT C T A G ATTTAAGAAGGAGATATACATATGGTGAGCAAAGGCGAAGAACTG

### Infection model

*G* *. mellonella* larvae were obtained from Reptilienkosmos.de (Schwalmtal, Germany) and selected for no visible signs of melanization, pupation, or any other illnesses before being infected. At the time of infection each larva weighed approximately 250 mg. Bacterial strains were grown overnight and the indicated amount of bacteria, suspended in 5 µl Dulbecco’s PBS (PAA, Pasching, Austria), was injected into the hindmost left proleg of the larvae using a 10 µl syringe (Hamilton, Bonaduz, Switzerland). As controls, non-injected and PBS-injected animals were included in each experiment. Groups of 16 identically treated larvae were placed in a 100 mm petri dish and incubated at 37° C. Individual larvae were scored regularly over the course of the experiment for signs of melanization and for viability using their reflex in response to contact. Data are expressed as percent survival, and are the representative results of at least three independent experiments.

### Flow cytometry

Larvae were first homogenized in 2 ml PBS using a sterile pipette tip. After vigorous pipetting, each lysate was cleared of coarse debris by centrifugation (500 *x* g, 10 min). A 1:100 dilution of the supernatant in 1% paraformaldehyde in PBS was then analyzed using a FACSAria III (BD, Heidelberg, Germany) cell sorter equipped with 488 nm and 561 nm lasers. Two-color analyses according to Bumann [34] were applied to discriminate between cellular debris and *Salmonella* expressing the fluorescent proteins SFGFP and TagRFP-T, respectively. For SFGFP detection, 488 nm excitation in combination with two detectors and the two filters, 502LP+530/30 and 555LP+586/15, was used. For TagRFP-T detection, 561 nm excitation in combination with two detectors and the two filters, 582/15 and 685LP+710/50, was used. Data acquisition was performed using FACS DIVA v 6.1.3 software (BD).

### Data analysis

Data were analyzed and plotted using Prism 5 software (GraphPad Software Inc., La Jolla, CAL, USA). Flow cytometry data was analyzed and plotted using FlowJo v 9.4.7 (Tree Star Inc., Ashland, OR, USA).

## Results

### Infection of *G* *. mellonella* with *S* . Typhimurium and determination of LD_50_

**Figure 1:**
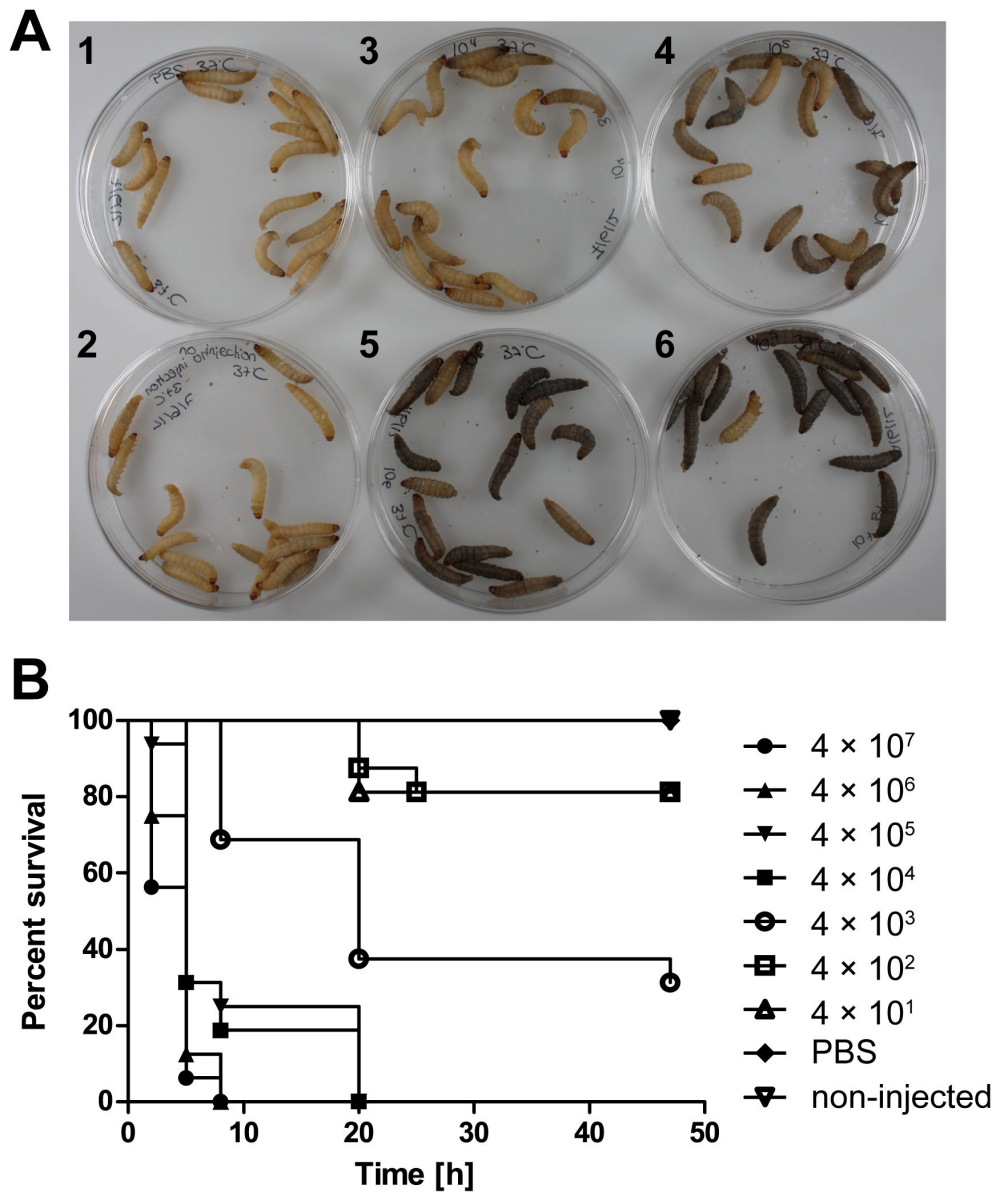
Dose-dependent killing of *G. mellonella* by *S.* Typhimurium NCTC 12023 WT. *G. mellonella* larvae were infected with increasing bacterial loads, ranging from 40 to 4 × 10^7^ bacteria, and incubated for up to 50 h at 37° C. (A) Deposition of melanin at 2.5 h p. i. was dependent on the number of bacteria injected (1) PBS control; 2) non-injected control; 3) 4 × 10^4^; 4) 4 × 10^5^; 5) 4 × 10^6^; 6) 4 × 10^7^). (B) The percent of larvae surviving was assessed after injecting different doses of *S*. Typhimurium WT as indicated. Increasing amounts of dark-colored and/or dead larvae were obtained in a dose-dependent manner. PBS: buffer control. Data as shown are the representative results of three independent experiments, for which similar outcomes were obtained.

In order to investigate the pathogenic potential of *Salmonella* towards arthropods, we studied the susceptibility of *G* *. mellonella* larvae to infection with *S* . Typhimurium strain NCTC 12023. To determine the parameters necessary for establishing infection by the pathogen, larvae were challenged with bacterial loads ranging from 40 to 4 × 10^7^ bacteria. Incubation was carried out at 37° C, the optimum temperature for *Salmonella* growth and mammalian physiology, and the larvae were monitored for up to 50 h to assess physical conditions, such as melanization and rates of survival. Melanization, a process resulting in the systemic deposition of the pigment melanin, is part of the innate immune response of Arthropoda to parasite challenge and can be used as an effective measure to evaluate insect health. Following infection with *Salmonella* , melanin production by *G* *. mellonella* became apparent only 2.5 h after injection of 4 × 10^5^ to 4 × 10^7^ bacteria (Figure 1A). Moreover, synthesis of melanin was triggered in a dose-dependent manner, eventually resulting in 100% dark-colored larvae at 20 h post inoculation using 4 × 10^7^ bacteria, as compared to moderate melanization in only 38% of larvae that received 4 × 10^4^ or fewer bacteria (data not shown). Non-injected, or PBS-injected larvae controls showed no signs of melanization (Figure 1A). In order to reflect a more physiological temperature for *G* *. mellonella* , infected larvae were incubated also at 27° C. Similar results to those described above were obtained under these conditions, although the progression of melanin production was notably slower (data not shown).

Following the onset of an immune response, larvae rapidly succumbed to *Salmonella* infection in a dose-dependent manner (Figure 1B). As seen for the deposition of melanin, PBS-injected or non-injected controls showed no symptoms, and 100% of control larvae survived (Figure 1A and 1B). Dose-dependent evaluation of *Salmonella* inoculi yielded an LD_50_ value of 3.6 × 10^3^ bacteria at 25 h post infection. Thus, further experiments were all performed using an inoculum of 4 × 10^4^ bacteria per larvae, which were followed for up to 48 h, to ensure that any minor differences in the pathogenic potential of the various *Salmonella* strains investigated would be observed (see below). As the onset of pupation occurred naturally after 40-48 h of incubation all experiments were concluded 48 h after bacterial challenge, at which point larvae were counted as either dead or alive.

It is widely accepted that changes occur in virulence factor expression by *S* . Typhimurium as it transits from logarithmic growth into stationary phase [35]. To evaluate the contribution of pre-induced virulence factors important for early colonization events such as invasion, adhesion or motility, we additionally compared the effects of using *S* . Typhimurium grown to late exponential or stationary phase for infection of *G* *. mellonella* . However, we did not observe any significant difference in the survival rates of larvae inoculated with either late-log invasive bacteria (3.5 h of sub-cultivation), or *Salmonella* that were grown overnight (20 h) to stationary phase (data not shown).

### Investigation of major virulence factors in the *Galleria* model

**Figure 2:**
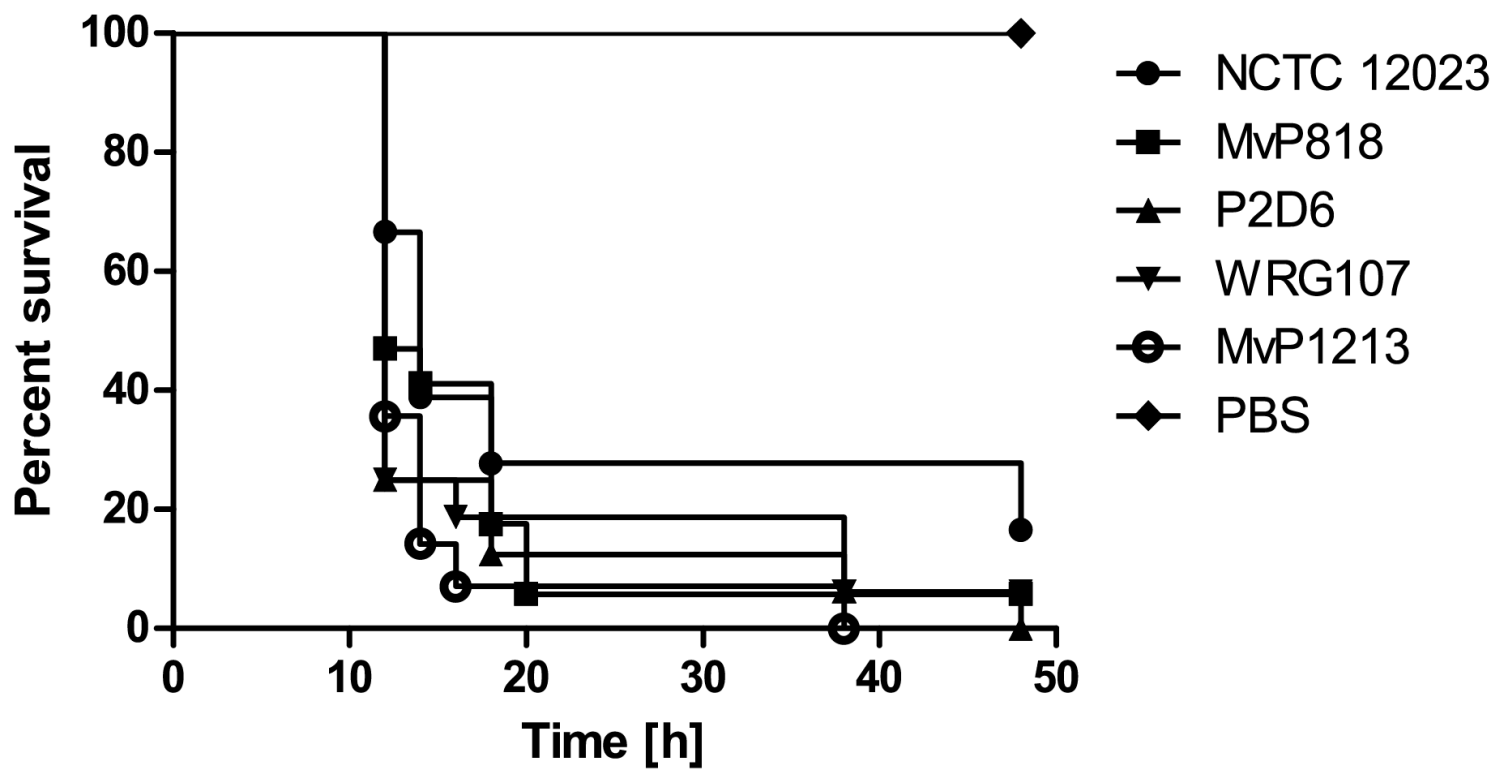
Survival of *G. mellonella* infected with *S.* Typhimurium NCTC 12023 WT or mutants deficient for known virulence factors. *G. mellonella* larvae were infected with 4 × 10^4^ *S*. Typhimurium NCTC 12023 WT and incubated for 48 h at 37° C. Likewise, larvae were challenged with the same amount of *Salmonella* mutant strains lacking a functional T3SS encoded by pathogenicity islands SPI-1 (MvP818), SPI-2 (P2D6), SPI-1 plus SPI-2 (WRG107) or the flagellar export ATPase gene, *fliI* (MvP1213). PBS injections were included as a negative control. Data as shown are the representative results of three independent experiments, for which similar outcomes were obtained.

The genus *Salmonella* possesses a variety of proteins, commonly referred to as virulence factors that are required for bacterial survival and/or proliferation within the host organism. A vast number of studies have identified various open reading frames (ORFs) that are transcribed from defined genomic regions known as Salmonella pathogenicity islands (SPI), which are essential for these host–pathogen interactions [2]. For instance, SPI-1 and SPI-2, each encoding genes for a type III secretion system (T3SS), were shown to be necessary for *in vitro* and *in vivo* infection of cell lines, *C. elegans*, mice or calves [10,36–38]. In order to investigate their contribution to *Galleria* infection, *G* *. mellonella* larvae were injected with *S* . Typhimurium mutant strains deficient in either a specific virulence factor, or an entire pathogenicity island (Table 1). Deletion of *fliI*, the gene encoding the flagella apparatus-associated ATPase, did not influence infectivity of the respective mutant strain MvP1213 (Figure 2). Likewise, strains disrupted in SPI-1, SPI-2 or both pathogenicity islands showed no attenuation when compared to the wild type (WT) strain NCTC 12023 (Figure 2).

### Assessing the impact of the PhoPQ regulon on *Galleria* infection

**Figure 3:**
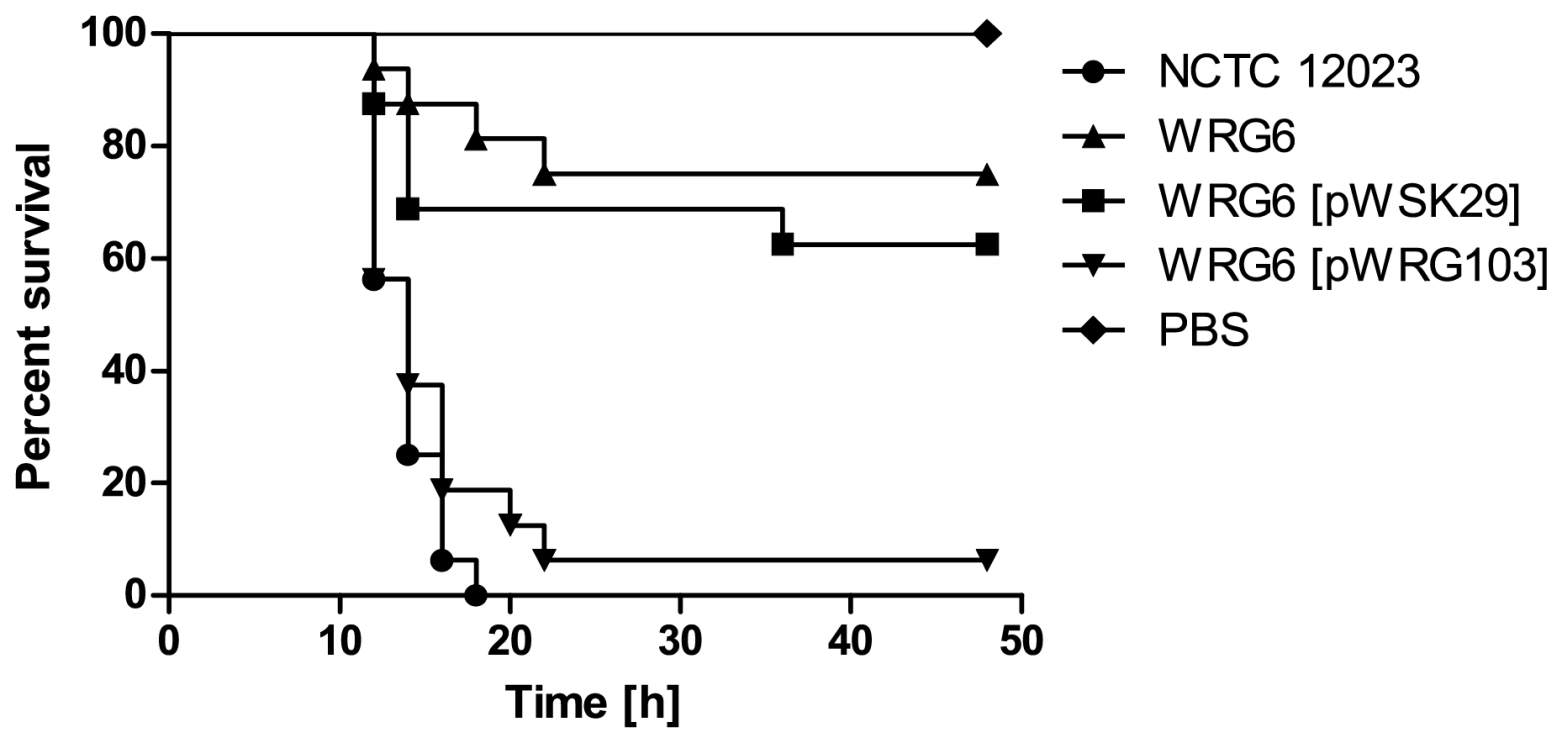
Survival of *G. mellonella* infected with *S.* Typhimurium NCTC 12023 WT and a Δ*phoQ* mutant strain. Survival of *G. mellonella* larvae was monitored for 48 h at 37° C after injection of *Salmonella* WT and the Δ*phoQ* deletion strain WRG6. Complementation of WRG6 was carried out *in trans* by transformation with the low-copy number plasmid pWRG103 expressing *phoQ*, or empty vector pWSK29. PBS: buffer control. Experiments were performed in triplicate using 16 larvae per group.

Previous studies have demonstrated that *Salmonella* of various serovars are capable of infecting the soil nematode *C. elegans*, where they reside in the lumen of the worm intestinal tract [13]. The ability to sense changes in the environmental surroundings with the two-component system PhoPQ is therefore a prerequisite for successful colonization by *S* . Typhimurium . PhoPQ has been shown to contribute to infectivity of this bacterium towards *C. elegans*, macrophages and mice alike [13,39]. Hence, we determined rates of survival of the *G* *. mellonella* larvae upon infection with a *phoQ* deletion mutant strain of *S* . Typhimurium . Deletion of the sensor kinase significantly reduced bacterial virulence in the *Galleria* model (Figure 3). While no larvae survived infection by WT bacteria, up to 75% of the arthropods were still alive at 24 h post challenge with the *phoQ* mutant strain WRG6 (Figure 3). This defect could be compensated for by trans-complementation experiments, whereby episomal introduction of *phoPQ* using the low-copy number vector pWRG103 resulted in the death of 94% of the larvae 24 h after challenge (Figure 3). By confirming that deletion of known virulence determinants, such as the conserved signaling pathway PhoPQ, attenuates the virulence of *S* . Typhimurium in this model these results support the utility in using *G* *. mellonella* as a model system for studying *Salmonella* pathogenesis.

### LPS is required for full virulence of *S* . Typhimurium in the *Galleria* infection model

**Figure 4:**
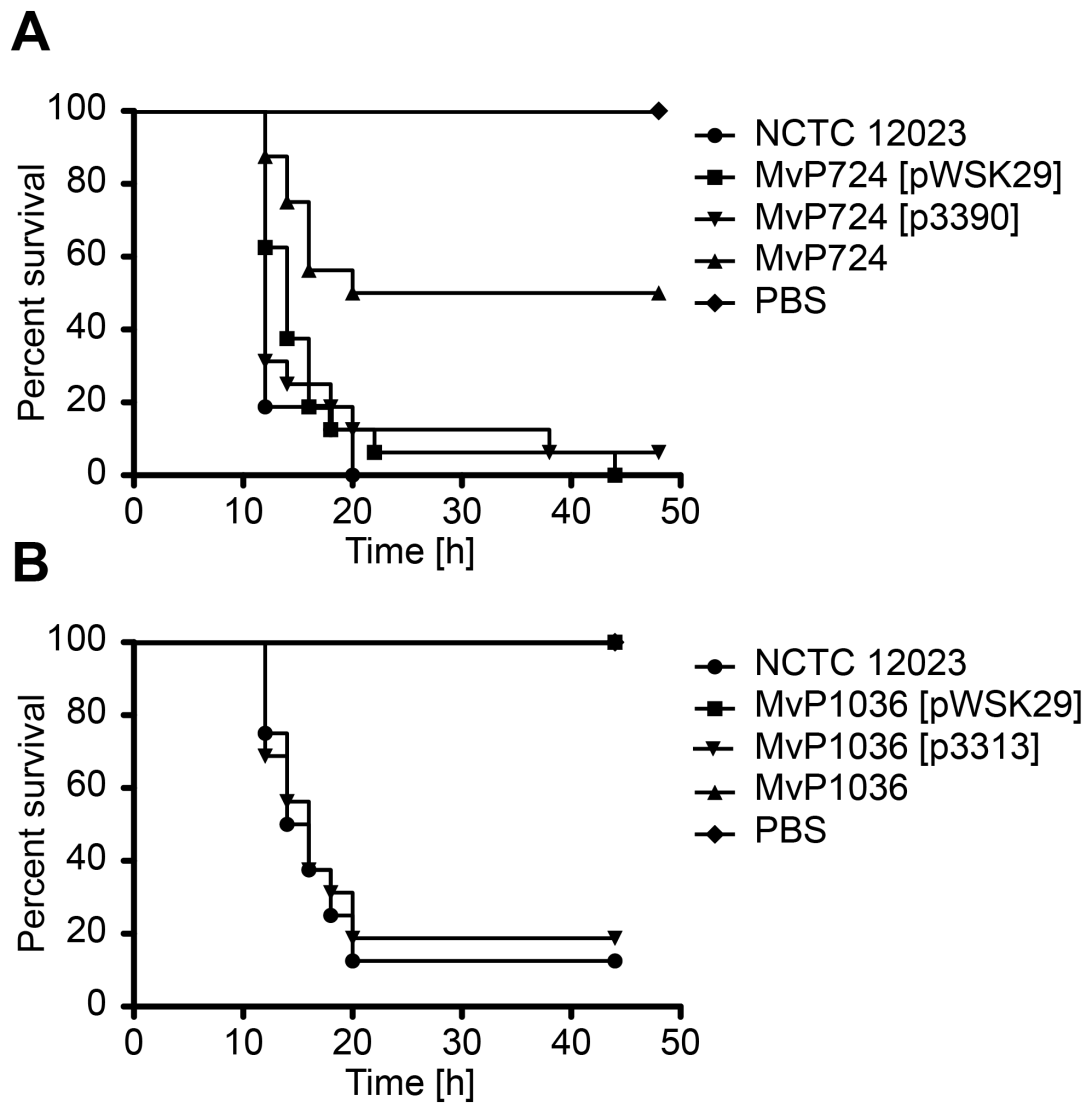
Effect of deletion of LPS-modifying enzymes on *Salmonella*-mediated killing of *G. mellonella*. Survival of *G. mellonella* was assessed after injection of 4 × 10^4^ *S*. Typhimurium NCTC 12023 WT or mutant derivatives lacking different LPS-modifying regulators and enzymes. (A) Comparison of WT with mutant strain MvP724, which lacks the regulators of gene expression *wzz*_ST_ */wzz*_fepE_. For complementation purposes, plasmid p3390 was transformed into MvP724, from which both *wzz*_ST_ and *wzz*_fepE_ were expressed under control of the *wzz*_ST_ promoter. Plasmid pWSK29 was transformed into the mutant strain as a vector-only control. (B) Impact of the O-antigen ligase, WaaL, on bacterial-mediated killing of *G. mellonella* larvae. Infection by the Δ*waaL* mutant MvP1036, with or without the empty vector (pWSK29), and its complemented derivative MvP1036 (p3313), was performed alongside WT injection at a total amount of 4 × 10^4^ bacteria per larvae. PBS: buffer control. Data as shown are the representative results of three independent experiments, for which similar outcomes were obtained.

PhoPQ signaling comprises a complex regulatory network that influences numerous cellular processes, including outer membrane modifications [40]. Because the LPS of *Salmonella* is detrimental to bacterial survival within host cells [12,41,42], we next analyzed whether truncation of this structure impacted *Salmonella* -induced killing of *G* *. mellonella* . Larvae were injected with the *wzz*_ST_/*wzz*_fepE_ double knockout mutant MvP724, which lacks the enzymes responsible for synthesizing long and very long modal length OAg, respectively [12,43]. Disruption of both ORFs results in a strain expressing a relatively short OAg (S–OAg). *G* *. mellonella* survival increased significantly upon truncation of the OAg (Figure 4A), while complementation of *wzz*_ST_/*wzz*_fepF_ *in trans* restored pathogenicity of the resulting *S* . Typhimurium strain to WT levels (Figure 4A). However, it must be noted that introducing the empty vector, pWSK29, used for *in trans* complementation also reconstituted virulence, albeit to a lesser extent than plasmid p3390, a pWSK29 derivative harboring both ORFs of *wzz*_ST_ and *wzz*_fepE_ (Figure 4A).

The contribution of LPS to *Salmonella* virulence was also examined by challenging *G* *. mellonella* larvae with the *waaL* (*rfaL*) mutant strain, MvP1036, which lacks the immunoresponsive oligosaccharide chain. As depicted in Figure 4B, deletion of the entire OAg completely abolished infectivity of this *S* . Typhimurium derivative. Twenty hours after inoculation only 12% of WT-infected larvae had survived bacterial challenge, whereas all larvae infected with the *waaL* mutant strain remained alive (Figure 4B). Complementation with long to very long modal lengths of OAg introduced by the low-copy number vector p3313 restored pathogenicity of mutant strain MvP1036 to 92% that of WT levels (Figure 4B). This was independent of carrying the empty vector alone, as transformation of the *waaL* mutant strain MvP1036 with pWSK29 did not influence virulence (Figure 4B).

Taken together, these data clearly demonstrate that *Salmonella* require an intact LPS with a specific modal length to resist the immune response, persist, and most likely multiply within the gastrointestinal tract of the arthropod *G* *. mellonella* .

### Competitive index analysis shows a role for LPS in bacterial replication within *G* *. mellonella*

**Figure 5:**
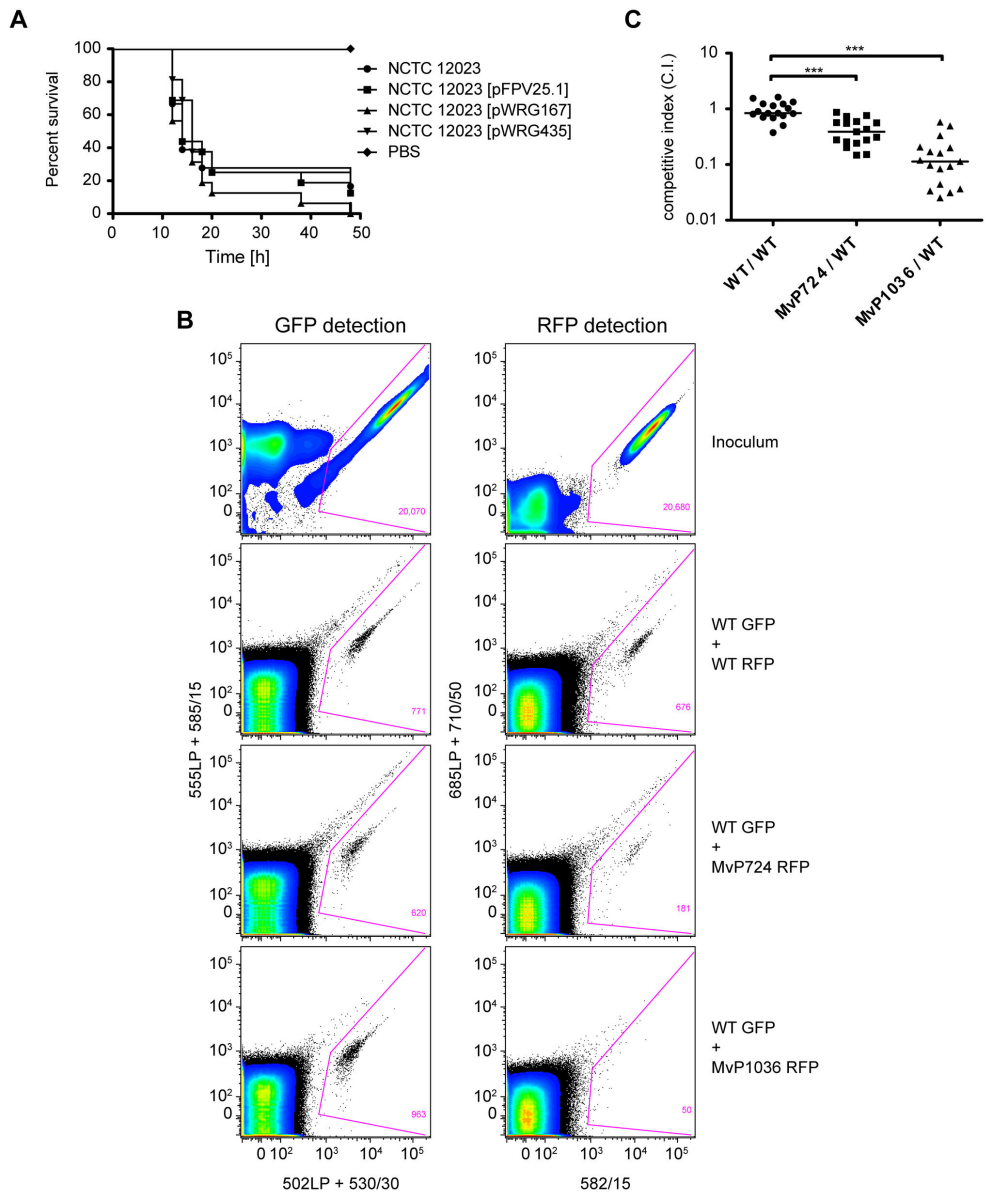
Impact of successive truncation of the O-antigen of *Salmonella* in the pathogenicity of these bacteria in competitive index experiments. *G. mellonella* larvae were injected with a suspension containing a total of 4 × 10^4^ bacteria. A 1:1 mixture of *S*. Typhimurium WT and each LPS-truncated mutant was used to inoculate the larvae, which were incubated for 6 h before larval lysates were analyzed by flow cytometry for bacterial quantification. Expression of either GFP from pFPV25.1 or pWRG167, or TagRFP‑T from pWRG435 was used to discriminate between WT and mutant strains, respectively. (A) The influence of *gfp* and *rfp* expression on bacterial-mediated killing of *G. mellonella* larvae was assessed using *S*. Typhimurium NCTC 12023 WT with or without the expression plasmids. PBS: buffer control. (B) Gating strategy and examples of bacterial cell counts as measured from homogenized larva tissue after co-infection with *Salmonella* WT and Δ*wzz*_ST_ */wzz*_fepE_ mutant (MvP724) or Δ*waaL* mutant (MvP1036). (C) Competitive index analysis of mixed infections involving WT/WT (*gfp*/*rfp*-tagged WT strain), MvP724/WT (*rfp*-tagged Δ*wzz*_ST_ */wzz*_fepE_ mutant and *gfp*-expressing WT) and MvP1036/WT (*rfp*-containing Δ*waaL* deletion mutant and *gfp*-tagged WT). One data point represents one individual larva. Horizontal bars indicate the median of data from three independent experiments. Statistical analysis by one-tailed Mann-Whitney ranked-sum test was done by comparing C.I. data of different co-infections as depicted. ***, *P* <0.0001.

*Salmonella* OAg length significantly contributes to *G* *. mellonella* mortality as shown above. To elucidate this effect in more detail we set out to establish a competitive index (C. I.) [44] model of *G* *. mellonella* infection, which would allow the relative fitness of a mutant strain to be assessed in comparison to the WT within an individual larva at much earlier time points than used previously. Two fluorescent marker proteins, GFP and RFP, were chosen to discriminate between mutant and WT strains in the co-infection. This methodology has been successfully used in a previous study analyzing the capability of different *Salmonella* mutants to replicate within cultured cells [45]. Since it is known that expression of fluorescent markers can significantly attenuate *Salmonella* virulence both *in vitro* and *in vivo* [46], we first tested whether WT bacteria expressing either GFP or RFP exhibited reduced virulence in the *G* *. mellonella* model when compared to a non-tagged WT strain. In single infections using 4 × 10^4^ bacteria per larva, no differences in pathogenicity were observed between the non-tagged WT, or WT strains expressing either SFGFP (from plasmid pWRG167) or TagRFP-T (pWRG435) following 48 h of infection (Figure 5A).

For each C. I. experiment, larvae were inoculated with 2 × 10^4^ each of the differentially tagged bacteria and followed for 6 h. We then used flow cytometry to determine the ratios between GFP-tagged WT bacteria and TagRFP‑T-tagged mutant strain in the inoculum, and in larval lysates 6 h post-infection. To discriminate between cellular debris of larval origin and the fluorescently labeled bacteria, samples were subjected to two-color flow cytometry for GFP as well as for RFP detection [34]. The gating strategy used for sample analysis is shown in Figure 5B. To calculate the C. I., the total numbers of fluorescent bacteria within these gates were used. Co-infection with differentially labeled WT *Salmonella* resulted in relatively equal amounts of GFP- and RFP-expressing bacteria in both the inoculum and larval lysates (mean C. I. = 0.962), proving that WT bacteria expressing either GFP or RFP are equally virulent in this model (Figure 5C). A co-infection using the RFP-tagged *wzz*_ST_/*wzz*_fepE_ double knockout mutant strain MvP724 together with GFP-tagged WT resulted in a mean C. I. of 0.43, indicating that significantly fewer mutant bacteria were present after 6 h of infection (Figure 5C). The colonizing ability of the RFP-labeled *waaL* mutant, MvP1036, at this time was also reduced, showing a six-fold reduction in bacterial numbers when compared to a GFP-labeled WT strain (mean C. I. = 0.165) (Figure 5C).

Taken together, these results confirm our observations from the single infection experiments, with the calculated C. I. reporting attenuation for both the *waaL* and the *wzz*_ST_/*wzz*_fepE_ *Salmonella* mutants just 6 h after infection of *G* *. mellonella* larvae.

## Discussion

### *Galleria* *mellonella* is susceptible to infection with *S* . Typhimurium strain NCTC 12023

The greater wax moth *Galleria* *mellonella* has been utilized as a novel model host for determining the virulence of a variety of bacterial pathogens. However, to date, data on infection of this caterpillar by the genus *Salmonella* has remained scarce. We herein demonstrate that these larvae are susceptible to *Salmonella* infection in a dose-dependent manner. Injection of about 3,600 *S* . Typhimurium NCTC 12023 cells was sufficient to cause the death of 50% (LD_50_) of the challenged larvae within 25 h of inoculation. LD_50_ scores for different pathogens are reported in the range from as little as 1 to exceeding 7 × 10^4^ bacteria per larva, depending on the experimental conditions used. While *P. aeruginosa* strain PA14 or selected strains of the *B. cepacia* complex were reported to exhibit an LD_50_ of less than 10 bacterial cells [21–23], *Acinetobater* *baumanii* , Group A Streptococci (GAS) and *L. monocytogenes* required 2 × 10^4^, 10^5^ and 10^6^ bacteria to cause 50% of the larvae to succumb to infection, respectively [19,47,48]. Our finding that *G* *. mellonella* is susceptible to *S* . Typhimurium infection indicates that this organism can be used as a novel model system for studying the pathogenic mechanisms of *Salmonella* .

### SPI-1 and SPI-2 are dispensable for *G* *. mellonella* infection by *S* . Typhimurium

In order to examine the virulence potential of certain pathogens in various hosts, researchers utilize deletion mutants with interruptions in known and putative virulence factors to study the role these specific determinants play in establishing and progression of disease. For example, depleting *P. aeruginosa* of its T3SS or important effector proteins such as the phospholipase ExoU renders this organism avirulent in a *Galleria* model killing assay [23]. Similarly, Tenor and colleagues also used *Salmonella* knockout strains to demonstrate the importance of both effector proteins and the entire SPI-1 in establishing intestinal colonization of *C. elegans* [10]. Deletion of one or two of the SPI-encoded T3SSs of *S* . Typhimurium also has a substantial impact on virulence during infection of mice [49,50]. Conversely, in this study we showed that the deletion of either SPI-1 or SPI-2 did not affect *Salmonella* virulence in a *Galleria* model of infection (Figure 2). However, since we only focused on data obtained by end point experiments, we cannot rule out a role for these two SPI-encoded T3SSs in pathogenicity of *S* . Typhimurium against *G* *. mellonella* at earlier time points of infection.

### *S* . Typhimurium requires PhoQ signaling and intact LPS for successful infection of *G* *. mellonella*

Although some established *Salmonella* virulence determinants do not appear essential for the successful colonization of the *Galleria* host, our study revealed that certain signal transduction pathways and outer membrane structures are important for larval infection. Deletion of *phoQ*, which encodes the membrane-spanning sensor kinase of the PhoP-PhoQ two-component system, severely attenuated virulence of *S* . Typhimurium NCTC 12023 in *G* *. mellonella* larvae (Figure 3). When ambient Mg^2+^-levels drop, as occurs in the transition from the environment to the host cell, PhoQ undergoes spontaneous autophosphorylation. Subsequent transfer of a phosphate residue to the response regulator, PhoP, leads to activation of gene transcription, stimulating a complex network of regulatory processes [51]. This PhoPQ regulatory system is essential for pathogenicity not only in *Salmonella* [39], but impacts the virulence of *Y. pestis* [52], *Shigella flexneri* [53] and the plant pathogen *Erwinia carotovora* subsp. *carotovora* [54]. Since all of these organisms thrive in quite distinct habitats, it is likely that this two-component system functions in the sensing of an appropriate environmental niche for colonization. Although *Salmonellae* usually reside inside a specialized vacuole within the host cell, it remains to be determined whether or not *S* . Typhimurium actively invades and replicates inside the hemocytes of *G* *. mellonella* .

In *Salmonella* , PhoPQ controls the expression of approximately 1% of all ORFs, many of which are involved in remodeling the bacterial cell envelope [39,55]. Included in this series of modifications is the incorporation of aminoarabinose into Lipid A, which reduces the negative net charge of the LPS and confers increased resistance to cationic antimicrobial peptides (CAMPs) such as polymyxin [40]. Moreover, PhoQ can be directly activated by CAMPs further contributing to the recognition of these antimicrobial molecules by *Salmonella* [56]. This might, at least in part, explain the reduced pathogenicity of S. Typmiurium *phoQ* mutants in our *Galleria* model. Since these larvae are capable of synthesizing a range of antimicrobial substances [57], the inability of these strains to acquire a resistant phenotype could severely impact bacterial viability inside the hemocoel.

Our investigations showed that truncation of the OAg had a dramatic effect on the ability of *S* . Typhimurium to kill the *Galleria* larvae. Shortening OAg chain length by deleting the regulatory genes *wzz*_ST_ and *wzz*_fepE_, reduced the pathogenic potential of the resultant mutant strain to 50% that of WT levels after 20 h of infection (Figure 4A). This effect was even more pronounced following disruption of the OAg liagse, WaaL, which rendered the mutant strain completely avirulent in our *Galleria* model of infection (Figure 4B). Truncation of the OAg has also been implicated in increased neutrophil-mediated killing, complement-mediated susceptibility, and phagocytosis of *Salmonella* by RAW264.7 macrophages [12,42,58]. Furthermore, Murray and colleagues demonstrated that a *wzz*_ST_/*wzz*_fepE_ double mutant of *S* . Typhimurium was highly attenuated in competition with the WT following intraperitoneally injection of mice, as well as in single infection analysis following oral administration [12]. This is consistent with our findings, whereby C. I. experiments clearly showed that the double mutant and the *waaL* deletion strain were outcompeted by WT bacteria in our *G* *. mellonella* model of infection. Interestingly, truncation of the LPS of *P. aeruginosa* results in increased secretion of effector proteins by the T3SS, and hence enhances lung injury [59]. However, this was not shown to be the case in *S* . Typhimurium , where reduced modal repeats within the LPS had no impact on T3SS function or intracellular replication by the bacteria [42]. Hölzer et al. identified another side effect of LPS truncation in *Salmonella* , in that the respective *waaL* mutant had impaired motility due to malfunction of the flagella assembly [42]. It is therefore feasible that the loss of virulence of the *waaL* strain in the *Galleria* model seen here is the result of the reduced ability of the bacteria to disseminate evenly throughout the larvae. However, as we did not observe attenuation of a non-motile *fliI* mutant strain, which lacks the flagella-associated ATPase, the mechanism through which loss of *waaL* attenuates virulence remains to be determined.

The presence of *Salmonella* -LPS is known to confer protection against the early epithelial immune response both *in vivo* and *in vitro* [41]. Likewise, intact LPS is required for the establishment of persistent infections, and causes gonadal cell death in *C. elegans* intestines [13]. In this *C. elegans* model of infection, the phenotypes of two distinct LPS mutants of *Salmonella* were comparable to that of the *phoPQ* deficient strain. We were able to replicate these findings using similar *phoQ*- and LPS- mutant strains in our *Galleria* model of *Salmonella* infection. Thus, prospective examinations using this novel model system should include detailed analysis of LPS-modifications in a PhoQ-dependent manner. This might reveal a positive correlation between PhoQ activation, signaling and subsequent LPS modification, which is important for *Salmonella* pathogenicity in various host backgrounds.

In conclusion, we demonstrated that *G* *. mellonella* represents a valuable alternative model for the investigation of *Salmonella* virulence determinants. The identification of LPS OAg length and PhoQ as factors important for the colonization of *G* *. mellonella* proves that there are an overlapping functions between virulence factors important for *Salmonella* pathogenesis in the wax moth, as well as in mammals. 
