# Correction: LPS Structure and PhoQ Activity Are Important for *Salmonella* Typhimurium Virulence in the *Gallleria* *mellonella* Infection Model

**DOI:** 10.1371/annotation/33579aee-7f41-4f86-8218-6497d469c28d

**Published:** 2013-09-20

**Authors:** Jennifer K. Bender, Thorsten Wille, Kathrin Blank, Anna Lange, Roman G. Gerlach, Junior Research Group

The affiliation shared by all author was incorrect. It should be: Junior Research Group 3, Robert Koch-Institute, Wernigerode, Germany.

The Current Address for author Anna Lange should be: Institute of Medical Microbiology and Hygiene, Interfaculty Institute of Microbiology and Infection Medicine (IMIT), University of Tübingen, Tübingen, Germany. 

