# Correction: LPS Structure and PhoQ Activity Are Important for *Salmonella* Typhimurium Virulence in the *Galleria* *mellonella* Infection Model

**DOI:** 10.1371/annotation/fb953ecb-3a75-4b3c-9f7c-eddb8e56fc4c

**Published:** 2013-10-16

**Authors:** Jennifer K. Bender, Thorsten Wille, Kathrin Blank, Anna Lange, Roman G. Gerlach, Junior Research Group

The term "*Galleria*" was misspelled in the article title. The correct title is: "LPS Structure and PhoQ Activity Are Important for *Salmonella* Typhimurium Virulence in the *Galleria mellonella* Infection Model." 

The correct citation is: Bender JK, Wille T, Blank K, Lange A, Gerlach RG (2013) LPS Structure and PhoQ Activity Are Important for *Salmonella* Typhimurium Virulence in the *Galleria mellonella* Infection Model. PLoS ONE 8(8): e73287. doi:10.1371/journal.pone.0073287 

